# *Lactobacillus rhamnosus* attenuates Thai chili extracts induced gut inflammation and dysbiosis despite capsaicin bactericidal effect against the probiotics, a possible toxicity of high dose capsaicin

**DOI:** 10.1371/journal.pone.0261189

**Published:** 2021-12-23

**Authors:** Wimonrat Panpetch, Peerapat Visitchanakun, Wilasinee Saisorn, Ajcharaporn Sawatpanich, Piraya Chatthanathon, Naraporn Somboonna, Somying Tumwasorn, Asada Leelahavanichkul

**Affiliations:** 1 Department of Microbiology, Faculty of Medicine, Chulalongkorn University, Bangkok, Thailand; 2 Department of Microbiology, Translational Research in Inflammation and Immunology Research Unit (TRIRU), Chulalongkorn University, Bangkok, Thailand; 3 Department of Microbiology, Faculty of Science, Chulalongkorn University, Bangkok, Thailand; 4 Microbiome Research Unit for Probiotics in Food and Cosmetics, Chulalongkorn University, Bangkok, Thailand; 5 Division of Nephrology, Department of Medicine, Faculty of Medicine, Chulalongkorn University, Bangkok, Thailand; University of Illinois at Chicago, UNITED STATES

## Abstract

Because of a possible impact of capsaicin in the high concentrations on enterocyte injury (cytotoxicity) and bactericidal activity on probiotics, *Lactobacillus rhamnosus* L34 (L34) and *Lactobacillus rhamnosus* GG (LGG), the probiotics derived from Thai and Caucasian population, respectively, were tested in the chili-extract administered C57BL/6 mice and *in vitro* experiments. In comparison with placebo, 2 weeks administration of the extract from Thai chili in mice caused loose feces and induced intestinal permeability defect as indicated by FITC-dextran assay and the reduction in tight junction molecules (occludin and zona occludens-1) using fluorescent staining and gene expression by quantitative real-time polymerase chain reaction (qRT-PCR). Additionally, the chili extracts also induced the translocation of gut pathogen molecules; lipopolysaccharide (LPS) and (1→3)-β-d-glucan (BG) and fecal dysbiosis (microbiome analysis), including reduced Firmicutes, increased Bacteroides, and enhanced total Gram-negative bacteria in feces. Both L34 and LGG attenuated gut barrier defect (FITC-dextran, the fluorescent staining and gene expression of tight junction molecules) but not improved fecal consistency. Additionally, high concentrations of capsaicin (0.02–2 mM) damage enterocytes (Caco-2 and HT-29) as indicated by cell viability test, supernatant cytokine (IL-8), transepithelial electrical resistance (TEER) and transepithelial FITC-dextran (4.4 kDa) but were attenuated by *Lactobacillus* condition media (LCM) from both probiotic-strains. The 24 h incubation with 2 mM capsaicin (but not the lower concentrations) reduced the abundance of LGG (but not L34) implying a higher capsaicin tolerance of L34. However, *Lactobacillus rhamnosus* fecal abundance, using qRT-PCR, of L34 or LGG after 3, 7, and 20 days of the administration in the Thai healthy volunteers demonstrated the similarity between both strains. In conclusion, high dose chili extracts impaired gut permeability and induced gut dysbiosis but were attenuated by probiotics. Despite a better capsaicin tolerance of L34 compared with LGG *in vitro*, L34 abundance in feces was not different to LGG in the healthy volunteers. More studies on probiotics with a higher intake of chili in human are interesting.

## Introduction

Capsaicin, a major active ingredient of chili, is responsible for the hot taste with several dose-related effects; ranging from the cell (or neuron) stimulation to the induction of cell death [1–3]. With the proper doses, capsaicin demonstrated several beneficial reactions, including anti-inflammation, anti-oxidants, anti-tumor, and immune stimulation [3–7]. Indeed, chili becomes one of the health-promoting diets [8]. However, consumption of extremely spicy food or high dose of capsaicin might induce some adverse effects. Accordingly, chili-induced diarrhea or dyspepsia is a common complication of spicy food consumption [9]. Although an inadequate time for water absorption and causes chili-induced diarrhea or loose stool due to capsaicin-induced gut-hypermotility (capsaicin irritating effect) is well-known [10, 11], diarrhea from other effects of capsaicin is possible. As such, the high doses of capsaicin activate inflammation and induce cytotoxicity (cell cycle arrest and apoptosis) in several cells [12–14] and capsaicin bactericidal activity [15] might selectively select some groups of gut bacteria lead to the fecal dysbiosis. Then, it is possible that too much consumption of chili might cause diarrhea from the direct enterocyte injury and gut dysbiosis. Although the pungency (spiciness) of Thai chili according to the Scoville scale, based on the concentration of capsaicinoid components is in the middle rank among all types of chili around the world [16, 17], Thai chili is an ingredient in nearly all Thai cuisines, even with fruits (mixing with salt and sugar). The frequent chili ingestion of Thai population might be associated with some unique features in their gut microbiota and the probiotics that are derived from Thai people might be more tolerance to capsaicin. Because of the possible similarity in gut microbiome within the population due to the influence of co-evolution (hosts and gut microbes) [18–21] and diets [22, 23], *Lactobacillus rhamnosus* L34 (L34; the probiotics derived from Thai microbiome [24–29]) might have a unique property against capsaicin. In parallel, *Lactobacillus rhamnosus* GG (LGG) are the commercially available probiotics that are derived from the Caucasian population with profound beneficial effects in several models [30–33]. Because animal models have been developed to study the effect of probiotics in various models [24–29], in this study we used C57BL/6 mice to test *Lactobacillus rhamnosus* in chili-induced gut injury model. Due to the possibility that L34 might be more tolerant against higher capsaicin concentrations than LGG, both strains of probiotics were tested in i) a mouse model with chili extract administration, ii) enterocytes (Caco-2 and HT-29 cell lines) and iii) the healthy volunteers.

## Materials and methods

### Animals and animal model

The Institutional Animal Care and Use Committee of the Faculty of Medicine, Chulalongkorn University, Bangkok, Thailand (SST025/2563) approved the animal care and use procedure in accordance with the US National Institutes of Health guidelines using 8-week-old male C57BL/6 mice (Nomura Siam International, Pathumwan, Bangkok, Thailand). All mice were housed in an animal facility that designed for the use of bacteria in mice under a 12:12 h light-dark cycle at animal center, Faculty of Medicine, Chulalongkorn University. Mice were kept in the same cage for the determination of several parameters in chili-administered model, while some mice were kept in different cages in each experimental group for the microbiota analysis.

To compare the result between 6 independent groups with one-way analysis of variance (ANOVA), we use G-power program to calculate sample size per group. Therefore, a minimum number of mice (8 mice per group) were used to obtain the proper statistical test and the significance was determined by *p*-value < 0.05. Then, a total of 48 mice were randomized into several groups for the determination of several parameters in chili-administered model and the feces from some mice were used for the fecal microbiome analysis. For investigating the effect of *Lactobacillus rhamnosus* in chili-administered model, mice were randomly divided into 6 groups: the control phosphate buffer solution (PBS) group (n = 8) without chili administration, the control *L*. *rhamnosus* 34 (L34) group (n = 8), the control *L*. *rhamnosus* GG (LGG) group (n = 8) and the chili-extract administered group treated with PBS (n = 8), L34 (n = 8), and LGG (n = 8) which received at 1 × 10^8^ colonies forming unit (CFU) of *Lactobacilli*. For chili extracts, 40 g of Thai chili was diluted in 250 mL of absolute ethanol before autoclave at 121°C for 15 min following a previous publication [34]. After that, the samples were subjected to filtration twice with Whatman filter paper (number 42) (GE Healthcare, Chicago, IL, USA) and dried in hot air oven at 55°C (Shel Lab, Cornelius, OR, USA) for 4 days before suspension by 60 mL sterile water. According to this protocol, the chili extract approximately contains 1 mg of capsaicin per 1 mL of the solution [34]. Then, the chili extract at 0.5 mL (approximately 50 mg capsaicin/ dose) or phosphate buffer solution (PBS) control was orally administered in mice once a day at 8:00 AM. In parallel, the probiotics, including *Lactobacillus rhamnosus* L34 (L34) (Chulalongkorn University, Bangkok, Thailand) or *Lactobacillus rhamnosus* GG (LGG) (Mead-Johnson, Evansville, IN, USA) at 1 × 10^8^ colonies forming unit (CFU) in 0.3 mL PBS or PBS alone were orally administered once a day at 16:00 PM. Mice were observed and monitored daily for body weight, stool consistency and feces from each group of mice were collected before sacrifice for microbiome analysis. After 14 days administration, mice were euthanized with cardiac puncture under isoflurane anesthesia and mouse samples (blood, and colon tissue) were collected. Serum and colon tissue were snap frozen in liquid nitrogen and kept in -80°C before use. The stool consistency was semi-quantitatively evaluated as “the stool consistency index” using the following score; 0, normal; 1, soft; 2, loose and 3, diarrhea, as previously published [35].

### Serum cytokine and gut permeability analysis

Serum tumor necrosis factor (TNF)-α was determined by enzyme-linked immunosorbent assays (ELISA) (Invitrogen, Carlsbad, CA, USA). Gut permeability was determined by fluorescein isothiocyanate dextran (FITC-dextran) assay, serum lipopolysaccharide (LPS), serum (1→3)-β-d-glucan (BG) and immunofluorescent detection of tight junction proteins following previous publications [28, 36–38]. The spontaneous detection of LPS and BG, the molecular components from gut microorganisms (Gram-negative bacteria and fungi, respectively), in serum without systemic inflammation and the identification of non-intestinal absorbable molecules in serum after an oral administration indicate gut permeability defect [36–38]. Then, FITC-dextran, an intestinal non-absorbable molecule with molecular weight (MW) of 4.4 kDa, (Sigma-Aldrich, St. Louis, MO, USA), at 12.5 mg per mouse was orally administered at 3 h before serum FITC-dextran detection by a Fluorospectrometer (NanoDrop 3300; ThermoFisher Scientific, Wilmington, DE, USA). Serum LPS and BG was measured by HEK-Blue LPS Detection (InvivoGen, San Diego, CA, USA) and Fungitell (Associates of Cape Cod, Inc., East Falmouth, MA, USA), respectively. When the values of BG were lower than 7.8 pg/mL, the data were recorded as 0 due to being beyond the lower limit of the standard curve. Additionally, gut tight junction was determined using mouse colons that were prepared in Cryogel (Leica Biosystems, Richmond, IL, USA), cut into 5 μm thick frozen sections and stained with antibodies against occludin and zonula occludens-1 (ZO-1) with the green fluorescent secondary antibody (Alexa Fluor 488) (Life Technologies, Carlsbad, CA, USA). The fluorescent color was visualized and scored by ZEISS LSM 800 (Carl Zeiss, Germany). For cytokines in colon tissue, including TNF-α and interleukin (IL)-6, the samples were weighed, sonicated thoroughly using a Ultra-Turrax homergenizer (IKA, Staufen, Germany) in 500 μL of PBS, pH 7.4 containing protease inhibitor, centrifuged at 12,000 × g for 15 min at 4°C to separate the supernatant and the supernatant cytokine levels were measured using ELISA (Invitrogen). Furthermore, 10% formalin fixed paraffin-embedded colon sections were stained with Hematoxylin and Eosin (H&E) (Sigma-Aldrich) and analyzed for intestinal injury with the modified semi-quantitative score at 200× magnification by 2 pathologists in a blinded manner based on mononuclear cell infiltration (in mucosa and sub-mucosa), epithelial hyperplasia (epithelial cell in longitudinal crypts), reduction of goblet cells, and epithelial cell vacuolization in comparison with control are following scores; 0; leukocyte < 5% and no epithelial hyperplasia (<10% of control), 1; leukocyte infiltration 5–10% or hyperplasia 10–25%, 2; leukocyte infiltration 10–25% or hyperplasia 25–50% or reduced goblet cells (>25% of control), 3; leukocyte infiltration 25–50% or hyperplasia >50% or intestinal vacuolization, 4; leukocyte infiltration >50% or ulceration as previously published [26].

### Gene expression of tight junction proteins using polymerase chain reaction

To quantitatively determine the intestinal tight junction injury, gene expression of occludin and ZO-1 were determined with quantitative reverse transcription polymerase chain reaction (qRT-PCR) following a published protocol [39]. Briefly, total RNA was prepared from the colon tissue samples with an RNA-easy mini kit (Qiagen, Hilden, Germany) and was quantified by Nanodrop 1000 Spectrophotometer (Thermo Scientific). Total reverse transcribed RNAs were processed with a High-Capacity cDNA Reverse Transcription (Thermo Scientific) before performing by qRT-PCR with SYBR Green PCR Master Mix using QuantStudio6 Flex Real-time PCR System (Thermo Scientific). The primers for occludin were (forward; 5′-CCTCCAATGGCAAAGTGAAT-3′, reverse; 5′- CTCCCCACCTGTCGTGTAGT-3′) and for ZO-1 were (forward; 5′- GCAAGAGGAGTCCCTGACTG-3′, reverse; 5′-CGGCTCTGTCCTAACTCCAG-3′). The results were demonstrated in terms of relative quantitation of the comparative threshold (delta-delta Ct) method (2^-ΔΔCt^) as normalized by *β-actin* (an endogenous housekeeping gene) with the following primers (forward; 5’-CGGTTCCGATGCCCTGAGGCTCTT-3’ and reverse; 5’-CGTCACACTTCATGATGGAATTGA-3’).

### Fecal microbiome analysis

Feces from each mouse (0.25 g per mouse; 3 mice per group) from different cages in each experimental group were collected for the microbiota analysis following a previous report [40]. Of note, mice in the same groups were housed in different cages because co-housing might induce similar gut microbiota within the same cage. In short, metagenomic DNA was extracted from individual mice by DNeasy PowerSoil Kit (Qiagen, Maryland, USA). The quality and concentration of the extracted DNA were measured by agarose gel electrophoresis and nanodrop spectrophotometry. Libraries of the V4 hypervariable region of 16S rRNA gene were amplified by polymerase chain reaction (PCR) using Universal prokaryotic primers 515F (forward; 5′-GTGCCAGCMGCCGCGGTAA-3′) and 806R (reverse; 5′-GGACTACHVGGGTWTCTAAT-3′), modified with the Illumina adapter and Golay barcode sequences in Miseq300 platform (Illumina, San Diego, CA, USA). The raw sequences and operational taxonomic unit (OTU) were classified following Mothur’s standard operating platform [41, 42]. The non-metric multidimensional scaling (NMDS), the distance-based ordination method, was performed based on the Bray-Curtis dissimilarity. The 16S rDNA sequences in this study were deposited in an NCBI open access Sequence Read Archive database with accession number PRJNA776693.

### Human fecal samples

Feces of the healthy volunteers were collected at the King Chulalongkorn Memorial Hospital, Bangkok, Thailand following the approved protocol by the Ethical Institutional Review Board, Faculty of Medicine, Chulalongkorn University (IRB No. 130/62), according to the Declaration of Helsinki, with the written informed consent from each individual volunteer. The inclusion criteria of the volunteers were i) adults between 18–65 years old without neither underlying diseases nor any current medications and ii) ingestion of Thai chili containing diet at least 1 meal per day. The exclusion criteria were i) underlying diseases (hypertension, diabetes, liver diseases, and kidney injury), ii) any medications, including antibiotics and health supplements within 1 month of the recruitment, and iii) ingestion of any products containing probiotics (for example; Yogurt, Kimchi and pickled fish) within 2 weeks. Then, the volunteers were orally once daily administered with *L*. *rhamnosus* L34 (Greater Pharma co., Bang Phlat, Bangkok, Thailand) or *L*. *rhamnosus* GG (Mead-Johnson) at 1 × 10^9^ CFU/dose. To determine the abundance of fecal *Lactobacilli* of the healthy volunteer, real-time polymerase chain reaction (PCR) was performed on fecal contents at the baseline (3 days before probiotic administration; 0 time-point (D0)) and after several days of administration, including 3 (D3), 7 (D7) and 20 (D20) days, and after stop the probiotics for 3 days (D23) and 7 days (D30) to explore rate of the probiotic reduction. The total DNAs were extracted by a QIAamp fast DNA Stool Mini Kit (Qiagen, Hiden, Germany) following manufacturer’s instructions with the primers for variable regions of 16S rRNA gene sequence of *L*. *rhamnosus*; rham (forward; 5′-TGCATCTTGATTTAATTTTG-3′) and Y2 (reverse; 5′-CCCACTGCTGCCTCCCGTAGGAGT-3′) [43]. The amplicon was approximately 290 base pairs (bp) and the genome size of *L*. *rhamnosus* (also designated as LR ATCC 53103) was 3,005,051 bp [44]. Bacterial genome is approximately 1.98 × 10^9^ g/mol and contains 6.02 × 10^23^ molecules/mol. One bacterium corresponds to 3.3 fg of DNA. The constructive of standard curve was generated by the QuantStudio™ Design & Analysis Software v1.4.3 (Thermo Fisher Scientific) using 10-fold serial dilution (6.6 fg to 660 pg) with bacterial concentrations ranging from of 2 to 2 × 10^5^ bacteria. In parallel, the quantification of fecal bacteria was indicated by real-time PCR which represented by cycle threshold (Ct value). Real-time PCR was performed in a QuantStudio™ Design & Analysis Software v1.4.3 with primers are as followed; total Gram negative bacteria (*16S rRNA* Gram neg.) (forward; 5′-GGAGGAAGGTGGGGATGACG-3′, reverse; 5′-ATGGTGTGACGGGCGGTGTG-3′), *Klebsiella* (KP16) (forward; 5′-GCAAGTCGAGCGGTAGCACAG-3′, reverse; 5′-CAGTGTGGCTGGTCATCCTCTC-3′) [45], *Salmonella* (STM4497) (forward; 5′-AACAACGGCTCCGGTAATGAGATTG-3′, reverse; 5′-ATGACAAACTCTTGATTCTGAAGATCG-3′) [46], *Bacteroides* (Bac) (forward; 5′-GGCGCACGGGTGAGTAAC-3′, reverse; 5′-TGTGGGGGACCTTCCTCTC-3′) [47], *Bacteroides fragilis* (forward; 5′-CGGAGGATCCGAGCGTTA-3′, reverse; 5′-CCGCAAACTTTCACAACTGACTTA-3′), *Lactobacillus* (L341bp) (forward; 5′-AGCAGTAGGGAATCTTCCA-3′, reverse; 5′-CACCGCTACACATGGAG-3′) [48], *Akkermansia* (AM) (forward; 5′-CAGCACGTGAAGGTGGGGAC-3′, reverse; 5′- CCTTGCGGTTGGCTTCAGAT-3′) [49], and total fungi (ITS) (forward; 5′-TCCGTAGGTGAACCTGCGG-3′ and reverse; 5′-TCCTCCGCTTATTGATATGC-3′) [50].

### Cell viability test of capsaicin-activated enterocytes

Because of the known cytotoxicity of capsaicin [12, 13] (a main substance responsible for the hot and spicy taste in the chili), capsaicin (305.41 g/mol molecular weight; Sigma-Aldrich, St. Louis, MO, USA) were tested with enterocytes (Caco-2 and HT-29 cell lines). As such, the human colorectal adenocarcinoma cells, Caco-2 (ATCC HTB-37) and HT-29 (ATCC HTB-38), from the American Type Culture Collection (Manassas, VA, USA) were maintained in supplemented Dulbecco’s modified Eagle medium (DMEM) and McCoy’s 5a modified medium, respectively, at 37°C under 5% CO_2_ and sub-cultured before use in the experiments. Then, capsaicin in the different concentrations (0.02, 0.2 and 2 mM) was incubated with the enterocytes for 24 h before the determination of cell viability using tetrazolium dye 3-(4,5-dimethylthiazol-2-yl)-2,5-diphenyltetrazolium (MTT) solution (Thermo Fisher Scientific, Rockford, IL, USA) [38]. The activated cells were incubated with 0.5 mg/mL of MTT solution for 2 h at 37°C in the dark and diluted by dimethyl sulfoxide (DMSO; Thermo Fisher Scientific) before measurement with a Varioskan Flash microplate reader at absorbance of optical density (OD) 570 nm.

### Proinflammatory activation in enterocytes

Because capsaicin induces diarrhea [9] possibly through capsaicin-induced gut hypermobility [10, 11] and cytotoxicity [12, 13] (especially with the presence of microbial molecules), capsaicin alone or with lipopolysaccharide (LPS), a major component of Gram-negative bacteria in gut, with or without (1→3)-β-d-glucan (BG), a major component of fungi in gut, were incubated with the enterocytes with and without the *Lactobacillus* condition media (LCM). For LCM preparation, *L*. *rhamnosus* L34 or LGG at an OD600 of 0.1 were incubated anaerobically for 48 h before supernatant collection by centrifugation and filtration (0.22-μm membrane filter) (Minisart; Sartorius Stedim Biotech GmbH, Göttingen, Germany). After that, cell-free supernatant of the samples (500 μL) was concentrated by speed vacuum drying at 40°C for 3 h (Savant Instruments, Farmingdale, NY), resuspended in an equal volume of DMEM or McCoy’s 5a modified medium for testing in Caco-2 or HT-29 cells, respectively, and stored at -20°C until use. Then, capsaicin (Sigma-Aldrich) at 0.02 mM alone or with LPS from *E*. *coli* O26:B6 (Sigma-Aldrich) at 100 ng/mL with or without BG, using whole glucan particle (WGP) that was purified from *Saccharomyces cerevisiae* (Biothera, Eagan, MN), at 100 μg/mL were incubated with the enterocytes with or without 5% (vol/vol) LCM (each strain) (the total volume was adjusted into 200 μL/well by the culture media) for 24 h before determination the level of IL-8 by using a Human CXCL8/IL-8 ELISA kit (Quantikine immunoassay; R&D Systems, Minneapolis, MN, USA) according to the manufacturer’s instructions.

### Transepithelial electrical resistance (TEER) and enterocyte permeability

The integrity of monolayer enterocytes in different conditions was determined by TEER using Caco-2 cells, but not HT-29 cells due to a limited ability of monolayer growth of HT-29 cells [51]. Caco-2 cells (ATCC HTB-37) at 5 × 10^4^ cells per well were seeded onto the upper compartment of 24-well Boyden chamber trans-well plate using DMEM-high glucose supplemented with 20% fetal bovine serum (FBS), 1% HEPES, 1% sodium pyruvate, and 1.3% Penicillin/Streptomycin for 15 days with daily media replacement to establish the confluent monolayer. After that, capsaicin (Sigma-Aldrich) at 0.02 and 0.2 mM with or without 5% (vol/vol) LCM of *L*. *rhamnosus* L34 or LGG were incubated at 37°C under 5% CO_2_ for 24 h. Subsequently, TEER was measured by an EMOM^2^ Epithelial Voltohmmeter (World Precision Instruments Inc., Sarasota, FL, USA) by placing the electrodes in supernatant at basolateral and apical chamber. The TEER value in media culture without cells was used as a blank and was subtracted from all measurements. The unit of TEER was ohm (Ω) × cm^2^. In parallel, 5 μL of FITC-dextran (4.4 kDa) (Sigma-Aldrich) at 10 mg/mL was added to the apical side of the trans-well chamber with 24 h stimulated Caco-2 cells. Then, FITC-dextran from the basolateral side of the trans-well plate was measured at 1 h after incubation using Fluorospectrometer (NanoDrop 3300) (ThermoFisher Scientific) as modified from the published protocols [52–54]. The concentration of FITC-dextran from the basolateral side represents the severity of permeability defect of Caco-2 cells.

### Capsaicin bactericidal effect against the probiotics

Due to the possible bactericidal activity of capsaicin against the probiotics [15], capsaicin (Sigma-Aldrich) at 20 mM in different concentrations (0.2, 2, and 20 mM) or medium control (De Man, Rogosa and Sharpe; MRS) were incubated with *L*. *rhamnosus* L34 (Chulalongkorn University) or *L*. *rhamnosus* GG (Mead-Johnson) at 3.0 × 10^7^ CFU/mL for 24 h before determination of bacterial abundance as previously described [55]. Briefly, *L*. *rhamnosus* at an OD600 of 0.1 (3.0 × 10^7^ CFU/mL) in MRS broth with or without capsaicin (Sigma-Aldrich) at 20 mM were incubated anaerobically for 24 h. After incubation, the optical density of each culture was determined at 600 nm (OD600) by spectrophotometer (Bio-Rad Smart Spec Plus, Bio-Rad Laboratories Inc, Hercules, CA, USA) to calculate bacterial number, and then 10-fold serially diluted in MRS broth, and cultured as stated previously for 48 h for bacterial enumeration.

#### Extracellular flux analysis

Because of the known influence on cell energy of capsaicin in cancer cells [56], the energy metabolism profiles of enterocytes (HT-29 cells) [57] with the estimation of glycolysis and mitochondrial oxidative phosphorylation through extracellular acidification rate (ECAR) and oxygen consumption rate (OCR), respectively, were performed by the Seahorse XF Analyzers (Agilent, Santa Clara, CA, USA) as previously described [28, 29, 58–62]. In brief, HT-29 cells (1 × 10^4^ cells/ well) were grown and stimulated with capsaicin in with McCoy’s 5a modified medium control, with or without LCM of *L*. *rhamnosus* L34 or LGG for 24 h in a Seahorse cell culture plate before replacing by Seahorse substrates (glucose, pyruvate, and L-glutamine) (Agilent, 103575–100) in pH 7.4 at 37°C for 1 h prior to the challenge with different metabolic interference compounds including oligomycin 1.5 μM, carbonyl cyanide-4-(trifluoromethoxy)-phenylhydrazone (FCCP) 1 μM, and rotenone/ antimycin A 0.5 μM according to the manufacturer’s instructions. The glycolysis data were analyzed by Seahorse Wave 2.6 software.

### Statistical analysis

Mean ± standard error of mean (SEM) was used for data presentation. The difference between groups was examined for statistical significance by one-way analysis of variance (ANOVA) followed by Tukey’s analysis or Student’s *t-*test for comparisons of multiple groups or 2 groups, respectively. All statistical analyses were performed with SPSS 11.5 software (SPSS, IL, USA) and GraphPad Prism version 9.0 software (La Jolla, CA, USA). A *p*-value of < 0.05 was considered statistically significant.

## Results

### Chili extracts induced loose stool and mild intestinal inflammation, partly through gut dysbiosis

**Fig 1:**
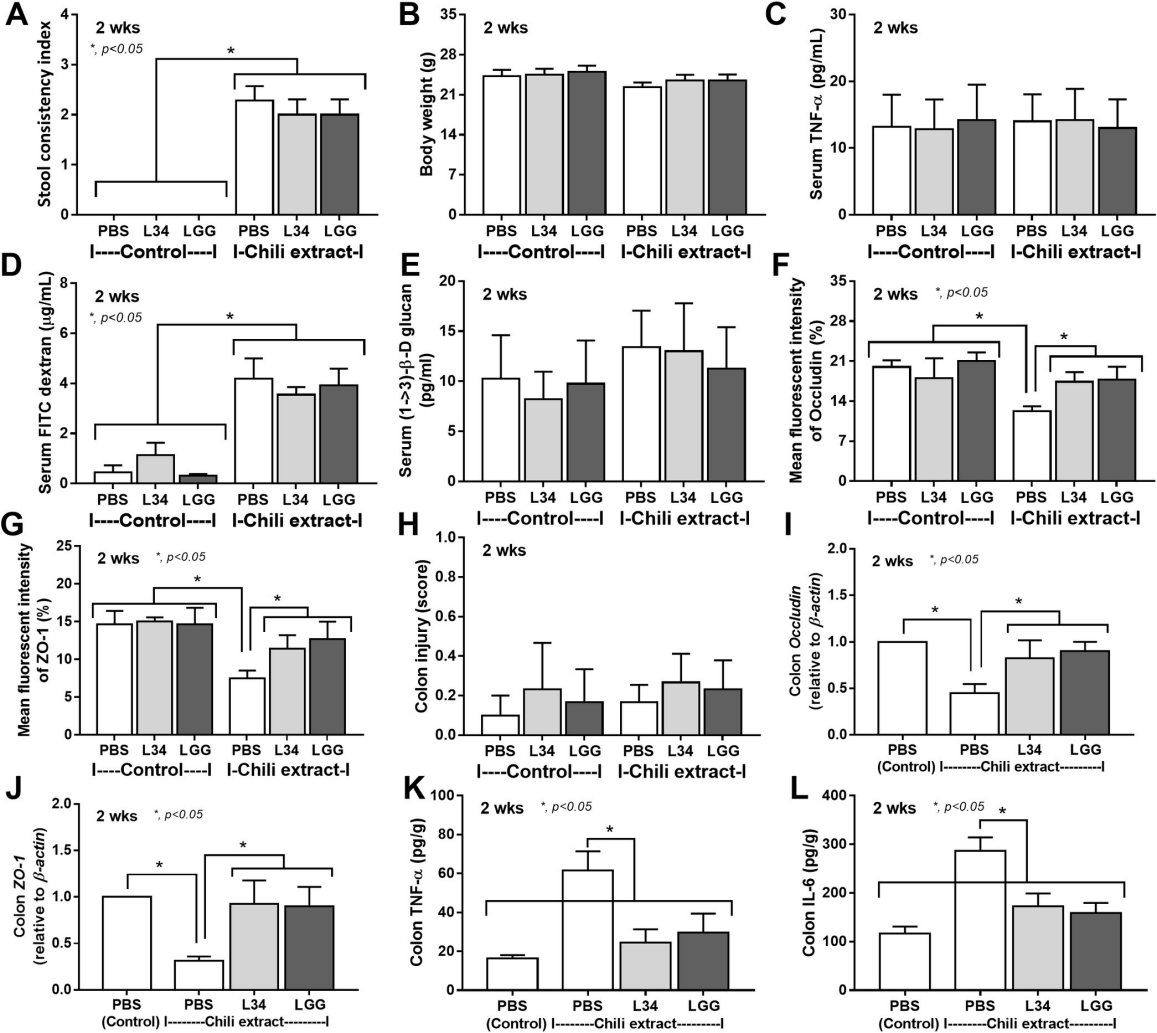
Characteristics of mice after administration by chili extracts or phosphate buffer solution (PBS) control with *Lactobacillus rhamnosus* L34 (L34) or *L*. *rhamnosus* GG (LGG) as determined by stool consistency index (A), body weight (B), serum TNF-α (C), gut barrier defect; FITC dextran assay, serum (1→3)-β-d-glucan (D, E) and injury of enterocyte tight junction molecules (occludin and ZO-1); mean fluorescent intensity (%) of occludin and ZO-1 (F, G), colon injury score (H), gene expression of occludin and ZO-1 on colon tissue (I-J), and inflammatory cytokines (TNF-α and IL-6) from colon tissue (K, L) are demonstrated (n = 8/ group). *, *p* < 0.05.

**Fig 2:**
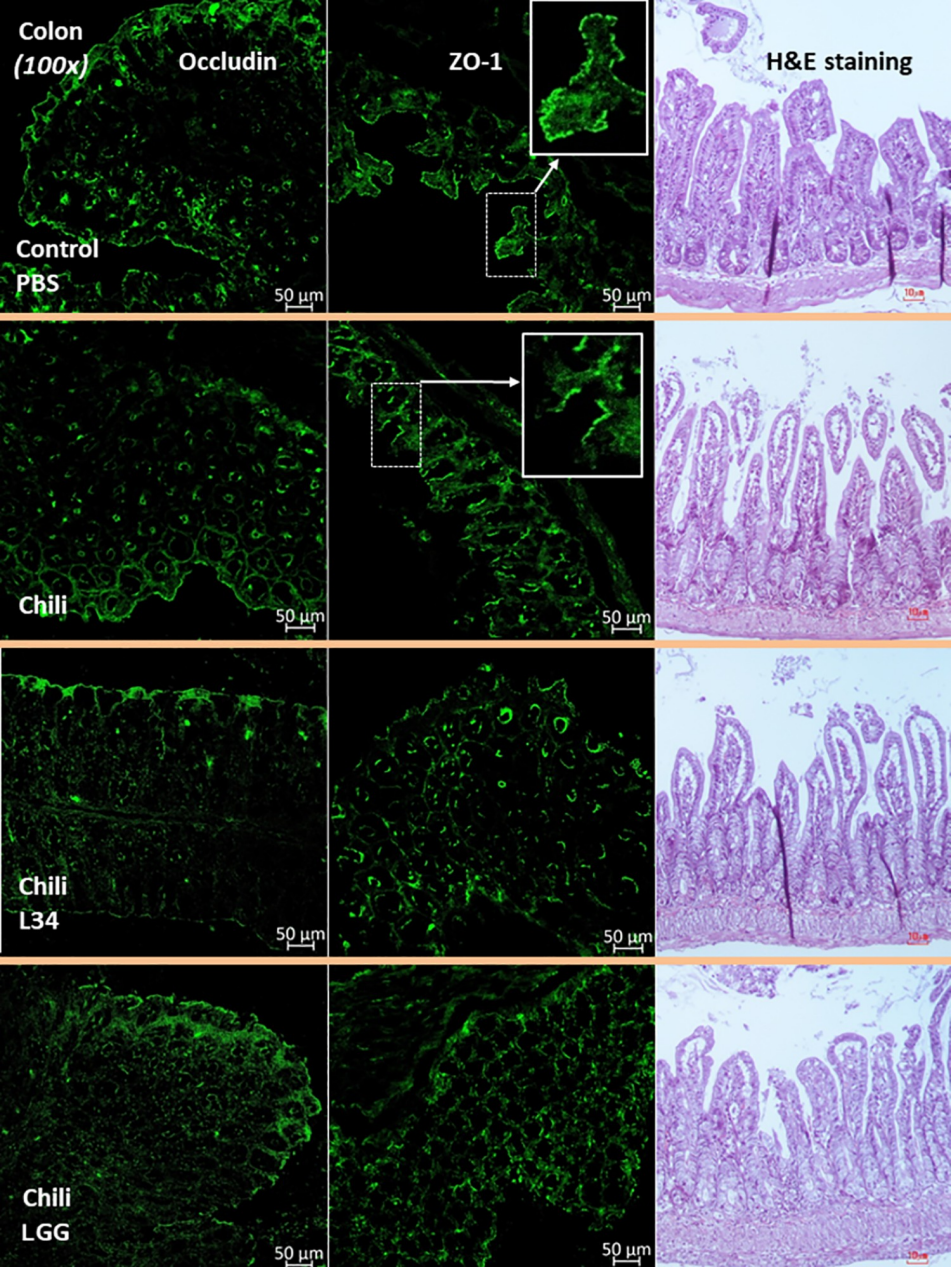
The representative pictures of colon from mice after administration by chili extracts or phosphate buffer solution (PBS) control or *Lactobacillus rhamnosus* L34 (L34) or *L*. *rhamnosus* GG (LGG) by fluorescent staining for occludin and ZO-1 together with Hematoxylin and Eosin (H&E) stained colon histology are demonstrated. The inset pictures were the high magnification of colons indicating a continuous (upper) and non-continuous (lower) ZO-1 staining from control PBS mice and chili-administered mice, respectively.

Chili extracts administration for 2 weeks in mice caused loose stool without an alteration in body weight and systemic inflammation (serum TNF-α) (Fig 1A–1C). However, the chili extracts induced gut permeability defect as indicated by FITC-dextran assay and the loss of intestinal tight junction molecules; occludin and ZO-1 (using fluorescent color staining and gene expression analysis), without the enhanced gut translocation of organismal molecules; (1→3)-β-d-glucan (BG) and lipopolysaccharide (LPS) (undetectable), nor colon histological injury (Figs 1D–1J and 2), indicating the limited severity of intestinal injury. Notably, LPS and BG are the major component of Gram-negative bacteria and fungi, respectively, which are the most and the second most abundance organisms, respectively, in gut [63]. Additionally, chili extracts also induced inflammatory cytokines (TNF-α and IL-6) in the colon tissue (Fig 1K and 1L), which supported a possible enterocyte toxicity from high dose of chili [2, 64]. The administration of *L*. *rhamnosus*, either L34 or LGG strains, attenuated the local inflammation (cytokines in colon tissue) (Fig 1K and 1L) and improved intestinal tight junction (Fig 1F, 1G, 1I and 1J) but not reduced the severity of gut barrier defect (Fig 1D) and not altered stool consistency (Fig 1A).

**Fig 3:**
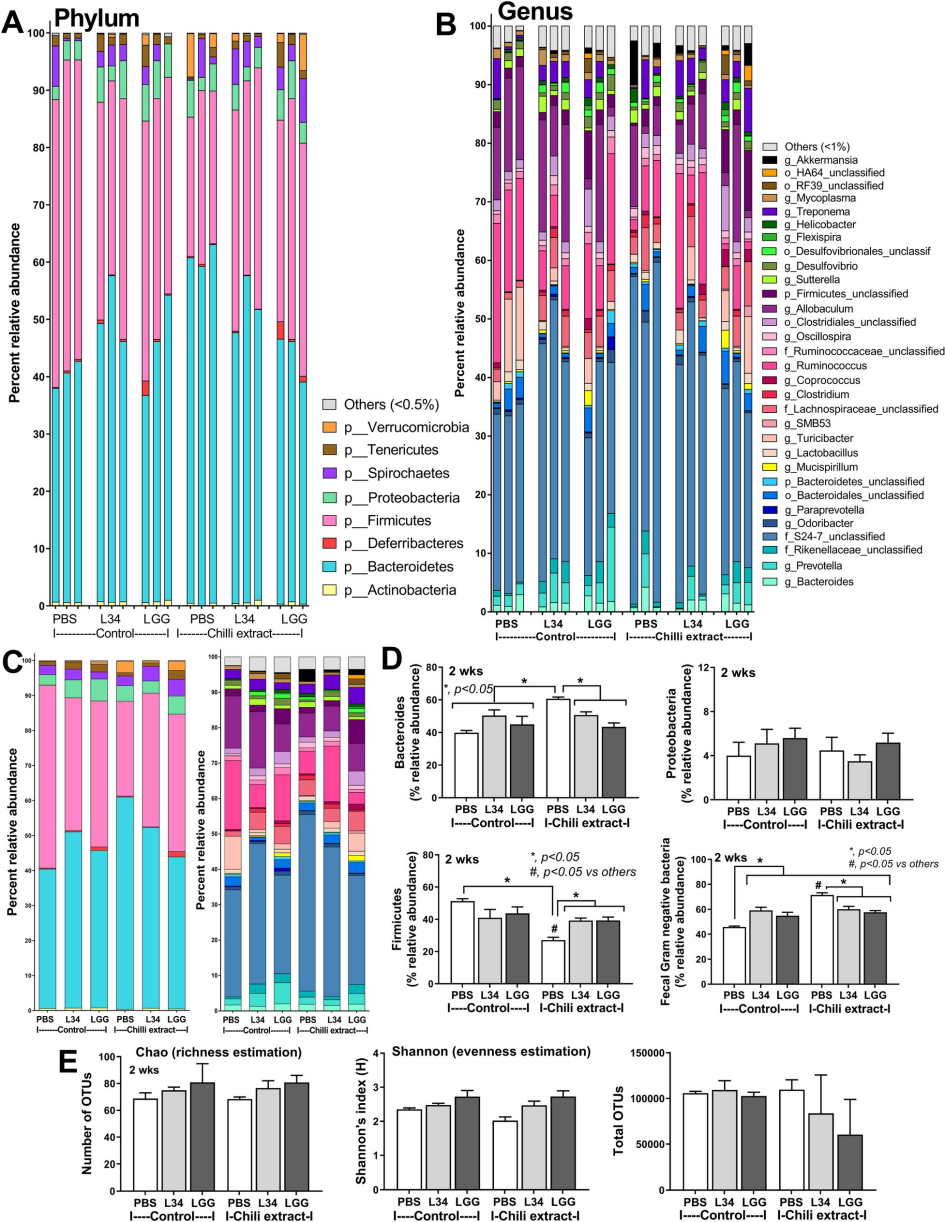
Gut microbiota analysis from feces of mice after administration by chili extracts or phosphate buffer solution (PBS) control with *Lactobacillus rhamnosus* L34 (L34) or *L*. *rhamnosus* GG (LGG) as determined by relative abundance of bacterial diversity at phylum and at genus level with the average abundance (A-C), relative abundance of bacterial diversity at phylum with graph presentation on Bacteroides, Proteobacteria, Firmicutes, and the fecal Gram-negative bacteria (D) and the alpha diversity by Chao1 index richness estimation and Shannon’s index evenness estimation with total operational taxonomic unit (OTUs) (E) are demonstrated. *, *p* < 0.05; ^*#*^, *p <* 0.05.

Because of bactericidal activity of capsaicin (a major chili active-component) [65], chili-induced gut inflammation might, at least in part, be due to gut dysbiosis. Indeed, chili extracts induced gut dysbiosis as indicated by increased Bacteroides, the Gram-negative anaerobes with a possible pathogenesis [51], decreased Firmicutes, the predominant organisms in healthy condition [51], and enhanced fecal total Gram-negative bacteria, a source of LPS (a potent pro-inflammatory inducer) in gut [60–62, 66], without an alteration in Proteobacteria, the pathogenic Gram-negative aerobes [51] (Fig 3A–3D). However, chili extracts did not alter neither the variety of microorganisms (Chao and Shannon indexes of alpha diversity) nor microbe abundance in gut as the values of the total OTUs (Fig 3E). Administration of *L*. *rhamnosus* L34 (L34) or *L*. *rhamnosus* GG (LGG) in the chili-extract administered mice reduced Bacteroides, decreased fecal Gram-negative bacteria and increased Firmicutes (Fig 3A–3D). Furthermore, the difference in fecal microbiome was demonstrated by a separation in non-metric multidimensional scaling (NMDS) of bacteria based on the species taxonomic level (S1A Fig). The major organisms (NMDS analysis) in control PBS group and chili-administered mice were *Streptococcus* spp. and *Butyricicoccus* spp., respectively, while various bacteria were indicated in the probiotic*-*administered groups implying an impact of probiotics on gut bacteria (S1A–S1C Fig).

### Capsaicin cytotoxicity on enterocytes and on probiotics, a possible impact of spicy food

**Fig 4:**
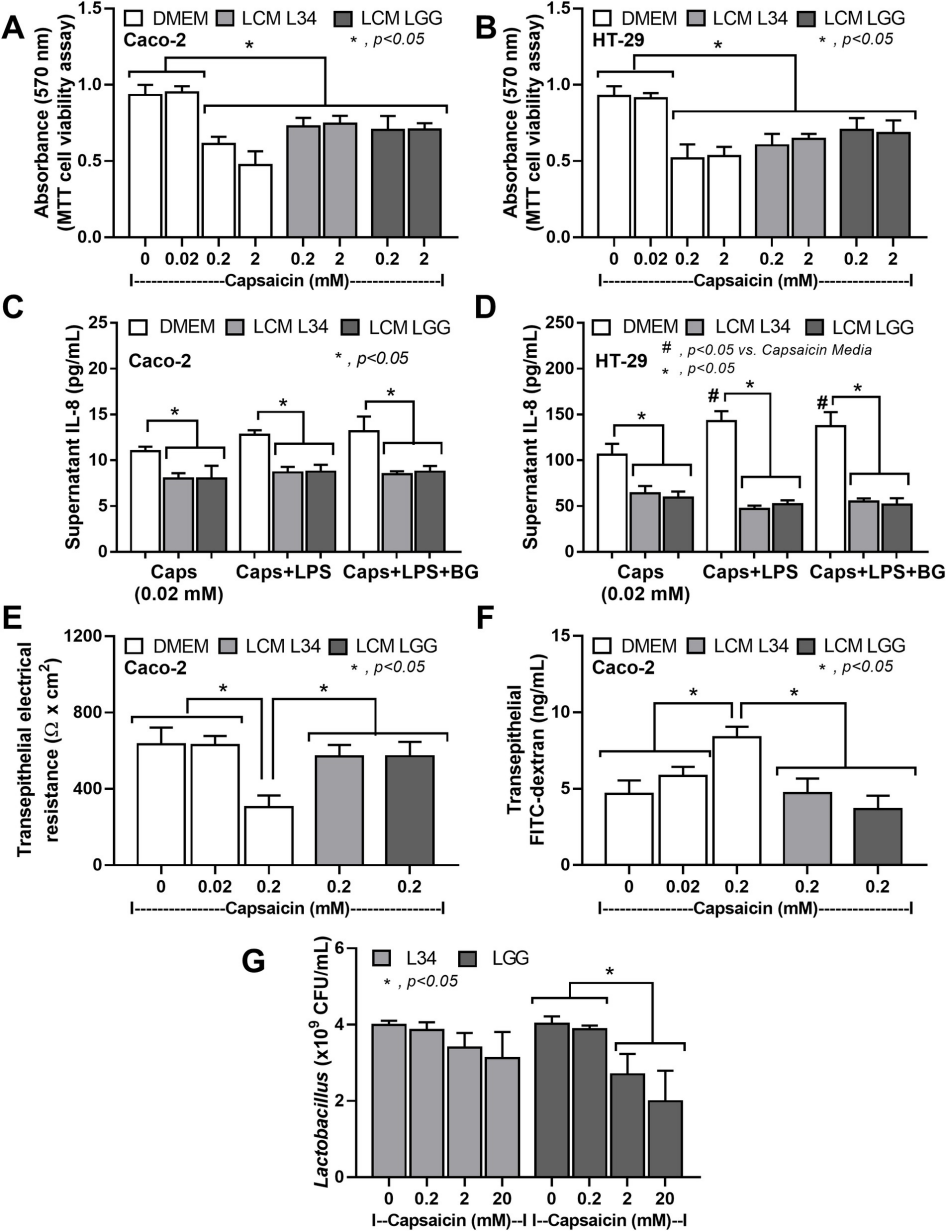
Capsaicin cytotoxicity on enterocytes and on probiotics. Cell viability of enterocytes (Caco-2 and HT-29 cells) with capsaicin (0.2 and 2 mM) in combination with or without the *Lactobacillus* condition media of *Lactobacillus rhamnosus* L34 (LCM L34) or *L*. *rhamnosus* GG (LCM LGG) for 24 h (A, B), supernatant IL-8 of the enterocytes (Caco-2 and HT-29) after 24 h incubation with capsaicin alone (Caps), Caps with lipopolysaccharide (Caps+LPS), Caps with LPS and (1→3)-β-d-glucan (Caps+LPS+BG) together with the LCM (C, D), the transepithelial electrical resistance (TEER) and transepithelial FITC-dextran of Caco-2 cells with capsaicin in combination with or without the LCM for 24 h (E, F) are demonstrated. Additionally, the abundance of *L*. *rhamnosus* L34 (L34) or *L*. *rhamnosus* GG (LGG) after 24 h incubation with capsaicin (0.2, 2, and 20 mM) (G) are also indicated. The results were from three independent experiments each in triplicate and expressed as the mean ± SEM. *, *p* < 0.05; ^*#*^, *p <* 0.05.

**Fig 5:**
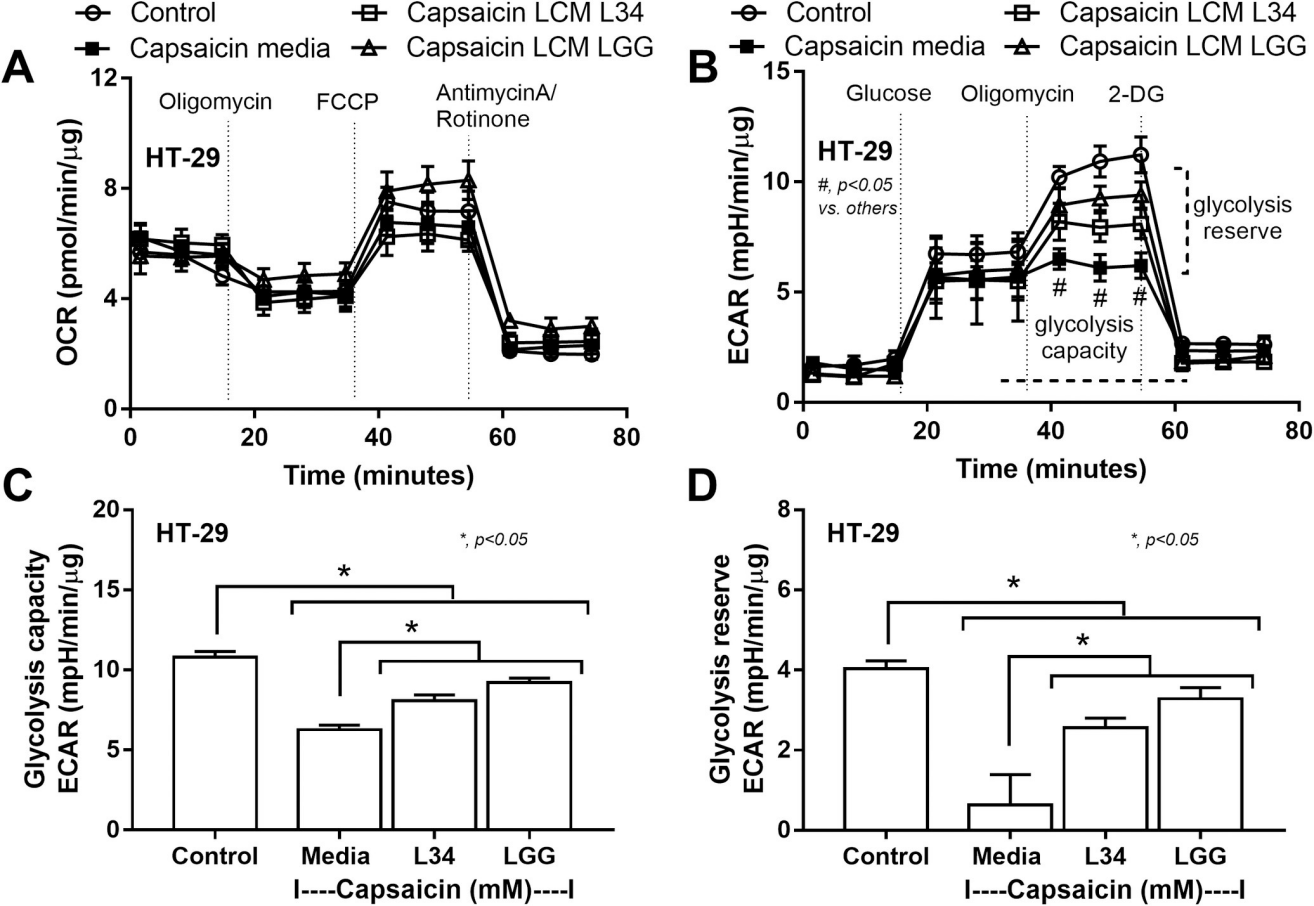
The extracellular flux analysis pattern of enterocytes (HT-29 cells) after 24 h incubation with tissue culture media control or stimulation with capsaicin (0.2 mM) in combination with or without the *Lactobacillus* condition media from *Lactobacillus rhamnosus* L34 (LCM L34) or *L*. *rhamnosus* GG (LCM LGG) as evaluated by oxygen consumption rate (OCR) of the mitochondrial stress test (mitochondrial oxidative phosphorylation) (A) and extracellular acidification rate (ECAR) of the glucose stress test (glycolysis pathway) (B) with the parameters of glucose stress test, including glycolysis capacity and glycolysis reserve (C-D) are demonstrated. Independent triplicate experiments were performed. *, *p* < 0.05.

Due to the well-known cytotoxicity and mild bactericidal activity of capsaicin [15, 55, 67, 68], capsaicin might directly induce enterocyte injury and reduce probiotics abundance. Indeed, capsaicin concentrations that higher than 0.2 mM reduced cell viability in both Caco-2 and HT-29 enterocytic cells which could be attenuated by *Lactobacillus* condition media (LCM) from both strains of the probiotics (L34 and LGG) (Fig 4A, 4B). Although capsaicin at 0.02 mM did not reduce cell viability (Fig 4A and 4B), this dose of capsaicin enhanced pro-inflammatory cytokine (IL-8) production that was also attenuated by LCM from both probiotics (Fig 4C and 4D). Notably, supernatant cytokines were non-detectable in enterocytes (Caco-2 and HT-29) in the control group (cell culture media alone). Additionally, capsaicin at 0.2 mM also worsened the integrity of enterocyte tight junction as indicated by the trans-epithelial electrical resistance (TEER) and trans-epithelial FITC-dextran (on Caco-2 cells) but LCM strengthened the integrity (Fig 4E and 4F). Although capsaicin at 0.2 and 2 mM reduced enterocyte permeability (Fig 4E and 4F) and cause enterocytic cell death (Fig 4A and 4B), respectively, capsaicin in both concentrations did not reduce the abundance of both L34 and LGG (Fig 4G). Capsaicin at a high dose (20 mM) decreased abundance of LGG, but not L34 (Fig 4G), implied a better capsaicin tolerance of L34. In parallel, capsaicin (at 0.2 mM), but not at 0.02 mM, decreased enterocyte cell energy as indicated by the reduced glycolysis activity, but not mitochondrial energy production (Fig 5A–5D), which supported a possible interference of cell energy metabolism by capsaicin as previously mentioned [56]. However, LCM from both L34 and LGG restored glycolysis activity (Fig 5A–5D) that might be associated with the reduced enterocyte inflammatory responses against capsaicin (Fig 4A–4F).

**Fig 6:**
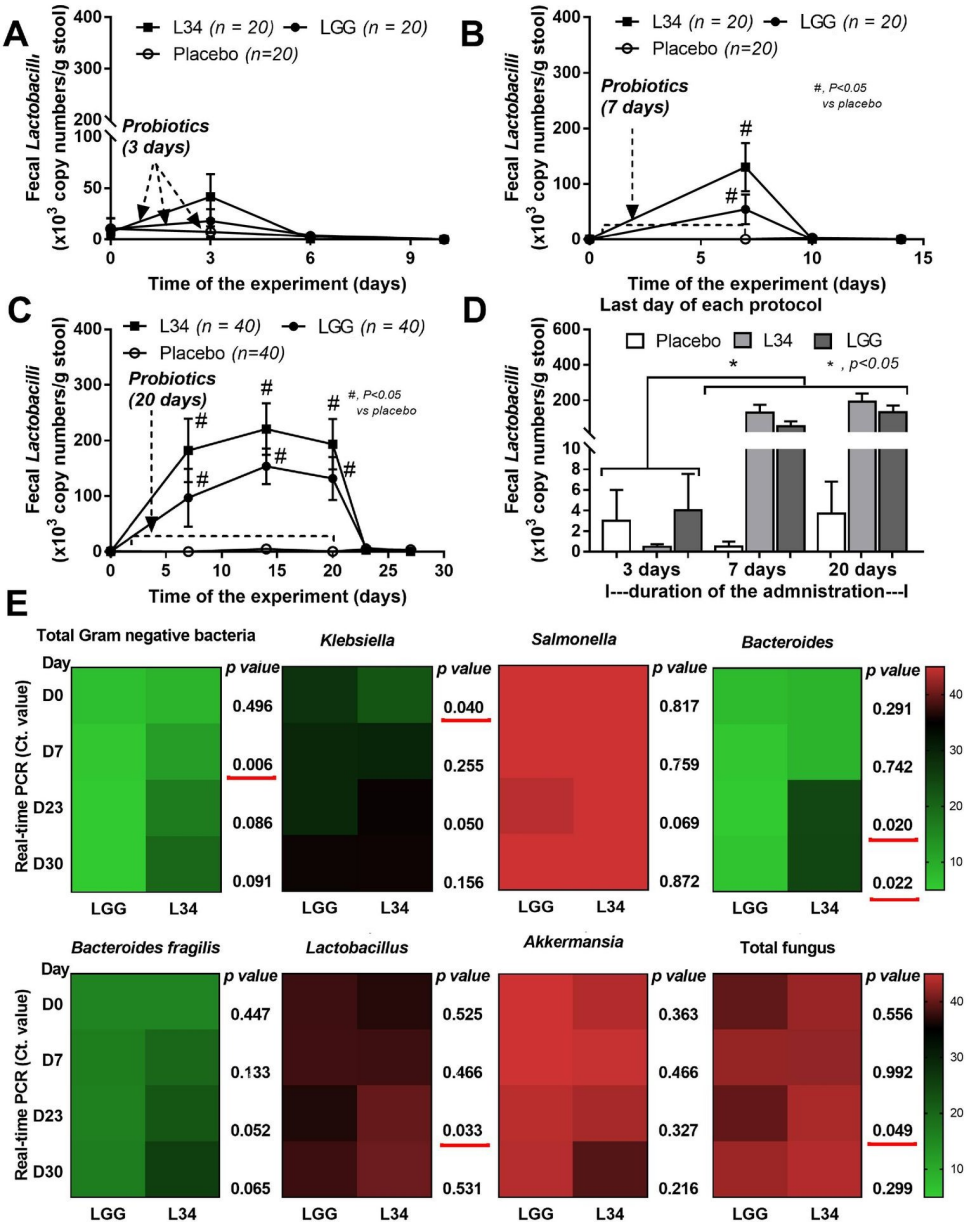
The abundance of fecal *Lactobacilli* from the human volunteers with placebo, *L*. *rhamnosus* L34 (L34) or *Lactobacillus rhamnosus* GG (LGG) after 3, 7 and 20 days of the administration using quantitative real-time polymerase chain reaction (PCR) in relative to standard curve of *Lactobacilli* (see method) (A-C) and fecal *Lactobacilli* at the last day of administration from the volunteers (for a better visualization) (D) are demonstrated. Additionally, the fecal abundance of several organisms using real-time PCR represented by cycle threshold (Ct. value) are also demonstrated. *, *p* < 0.05.

Because *L*. *rhamnosus* L34 is derived from Thai population and most of Thai cuisines compose of chili (even with some fruits), L34 might be a strain with a higher tolerance to chili than LGG (the probiotics derived from the Caucasian) [69]. Then, L34, LGG or placebo was administered in healthy Thai volunteers (Table 1) that consume Thai chili at least a meal per day. However, abundance of *Lactobacilli* in feces of the volunteers was not different between L34 and LGG after the administration for 3, 7, and 20 days (Fig 6A–6C). The *Lactobacilli* abundance in the volunteers with placebo was very low or non-detectable in the placebo group and the abundance in the probiotic-administered groups (more than 7 days) were higher than the placebo group but rapidly disappeared within 3 days after stop probiotics (Fig 6A–6D). The characteristics of *Lactobacilli* fecal abundance were similar between L34 and LGG (Fig 6A–6D), despite a better tolerance on the high dose capsaicin of L34 over LGG *in vitro* (Fig 4G). In some time-points of administration, fecal bacterial abundance (detected by real-time PCR) of the L34-administered volunteers, including *Klebsiella*, *Bacteriodes*, *Bacteriodes fragilis*, and total Gram-negative bacteria, were lower than the LGG group, while fecal abundance of *Lactobacilli* and total fungi in the L34-administered group were higher than the LGG group (Fig 6E). Further studies on the impact of heavy spicy foods or other diets on probiotics are interesting.

**Table 1:** Epidemiology of the volunteers.

Duration (number)		Female	Age (years)	Hct (%)	Scr (mg/dL)	ALT (U/L)	ALP (U/L)	Chili dose*
3 days	Placebo	9	39 ± 11	42 ± 3	1.09 ± 0.04	32 ± 5	55 ± 3	0.8 ± 0.3
(20 per	L34	8	41 ± 5	41 ± 5	1.21 ± 0.05	27 ± 10	47 ± 12	0.9 ± 0.2
group)	LGG	12	47 ± 11	45 ± 6	0.98 ± 0.04	35 ± 3	50 ± 7	0.8 ± 0.4
7 days	Placebo	9	42 ± 12	43 ± 5	1.08 ± 0.03	38 ± 4	39 ± 10	0.9 ± 0.2
(20 per	L34	11	39 ± 17	41 ± 3	1.01 ± 0.08	21 ± 4	42 ± 7	0.9 ± 0.5
group)	LGG	14	43± 12	44 ± 5	1.04 ± 0.07	33 ± 6	40 ± 11	0.8 ± 0.3
20 days	Placebo	14	40 ± 12	44 ± 3	1.05 ± 0.04	31 ± 5	45 ± 9	0.9 ± 0.2
(40 per	L34	10	42 ± 12	46 ± 7	1.07 ± 0.05	27 ± 7	41 ± 8	0.8 ± 0.3
group)	LGG	12	40 ± 15	45 ± 6	1.15 ± 0.03	32 ± 5	43 ± 5	0.7 ± 0.2

Hct, hematocrit; Scr, serum creatinine (mg/dL); ALT, alanine transaminase (U/L)

ALP, alkaline phosphatase (U/L); LGG, *L*. *rhamnosus* GG; L34, *L*. *rhamnosus* L34

*, tablespoon/ day.

## Discussion

The high dose of chili administration for 2 weeks induced mild enterocyte inflammation and gut permeability defect through chili-induced gut dysbiosis and capsaicin-induced enterocyte cytotoxicity, which could be attenuated by *Lactobacillus rhamnosus*. Despite several health benefits of capsaicin (a major beneficial component in chili) [70], the substance in a high dose (50 mg/day) might induce an adverse effect as indicated by gut barrier defect from the high dose of chili extracts. Here, 2 weeks administration of Thai chili extracts induced loose stool in all mice supporting the well-known capsaicin induced gut hyper-motility [10, 11]. Due to the inadequate time in the intestine for water absorption, gut hyper-motility causes loose stool and diarrhea. Additionally, the chili extracts also induced gut permeability defect that severe enough for the translocation of FITC-dextran, a non-gut absorbable with molecular weight (MW) 4.4 kDa, but was not severe enough for gut translocation of the larger pathogen molecules, including lipopolysaccharide (LPS; MW 10–100 kDa or higher) and (1→3)-β-d-glucan (BG; MW 6–600 kDa or higher) [63]. Notably, passive transport through the healthy intestinal tight junction normally allows gut translocation of only the molecules smaller than 0.6 kDa [63]. Despite the non-translocation of LPS and BG, there was a mild reduction of occludin and ZO-1, the tight junction molecules, after chili administration indicating a possible higher sensitivity of a fluorescent-based detection of gut barrier defect.

Although the anti-inflammatory and anti-oxidant effects of capsaicin are mentioned [6, 71, 72], the high dose of capsaicin induces neurogenic inflammation and cytotoxicity [73, 74]. Here, the dose-related enterocyte toxicity of capsaicin was indicated as the low dose capsaicin (0.02 mM) induced only enterocyte inflammation (IL-8 production) without an effect on cell viability, transepithelial electrical resistance (TEER), and cell energy. However, the pro-inflammatory effect of 0.02 mM capsaicin against enterocytes were enhanced by the presence of LPS and BG. With 0.2 mM capsaicin, there was a reduction in enterocytic cell viability, TEER, and cell glycolysis activity supporting several previous reports [1, 14, 56, 75–79]. Furthermore, capsaicin in the very high dose (2 mM) reduced enterocyte viability and demonstrated bactericidal effect on *L*. *rhamnosus* GG supporting cytotoxicity and bactericidal activity of capsaicin [14, 80–82]. Hence, gut barrier defect from the high dose chili extracts might be partly responsible from the direct enterocyte cytotoxicity of capsaicin that induced local inflammation (colon cytokines; TNF-α and IL-6) and loose stool.

High dose chili extracts induced intestinal barrier defect partly through fecal dysbiosis. The fecal dysbiosis is another common cause of intestinal tight junction defect and diarrhea [26] that might be partly associated with the loose stool after chili administration. Accordingly, the chili extracts induced gut dysbiosis as indicated by reduced Firmicutes, the highest abundant bacteria in healthy condition [83], and increased Bacteroides, bacteria with possible pathogenicity in some conditions [84], and enhanced total Gram-negative bacteria, the source of LPS in mouse feces. Although capsaicin in a lower dose (8 mg/kg/dose) increases Firmicutes and reduces Bacteroides in mice [85], high dose capsaicin (more than 0.33 mM) demonstrates bactericidal effect against some bacteria, including Firmicutes and Bacteroides [86], that might be responsible for chili-induced fecal dysbiosis are previously reported. As such, the non-metric multidimensional scaling (NMDS) analysis identified 2 different predominant bacterial clusters as following; i) *Streptococcus* spp. (Family; *Streptococcaceae*) in the control PBS group and ii) *Butyricicoccus* spp. (Family; *Clostridiaceae*) in the chili-administered group. Although both bacteria are possible beneficial microbes in Firmicutes group [87–89], the NMDS analysis demonstrated some differences between chili-administration and the control PBS group. Not only bactericidal effect of high dose capsaicin, the intestinal inflammation is also a factor that could directly induce fecal dysbiosis [90] and some of the beneficial microbes (such as *Butyricicoccus* spp.) might be enhanced to counteract with the dysbiosis. More exploration on *Butyricicoccus* spp. might be interesting. Meanwhile, NMDS plot among *L*. *rhamnosus* (either L34 or LGG) administered groups identified several bacteria in the different directions from the control PBS and the chili-administered groups, implying a possible impact of the probiotics.

On the other hand, there were similar benefits of *L*. *rhamnosus* GG and *L*. *rhamnosus* L34, despite the capsaicin bactericidal effect on *L*. *rhamnosus* GG. As such, the attenuation of gut barrier defect and fecal dysbiosis by several probiotics, including *L*. *rhamnosus*, is well-known [28, 29, 51]. Here, both *L*. *rhamnosus* L34 (L34; the Thai-derived probiotics) and *L*. *rhamnosus* GG (LGG; the commercially available Caucasian-derived probiotics) attenuated intestinal inflammation, tight junction defect, and fecal dysbiosis. While the NMDS fecal analysis clearly separated between the chili-administration and the control PBS groups, the analysis on feces from mice with probiotics could not be clearly separated to other groups. Both probiotics (L34 and LGG) increased Firmicutes, reduced Bacteroides, and decreased total Gram-negative bacteria when compared with the chili-extract administered mice without probiotics. Although several mechanisms are responsible for probiotics beneficial effects, the anti-inflammatory substances might be one of these mechanisms. Indeed, *Lactobacillus* condition media (LCM) from both L34 and LGG attenuated several effects of high dose capsaicin stimulation, including cell viability, pro-inflammation, and cell resistance (TEER) which might be associated with a preservation on the glycolysis activity. Indeed, cell energy status of the enterocytes is necessary for the maintenance of several cell activities [91, 92]. In parallel, 24 h incubation of 2 mM capsaicin reduced LGG abundance but no effect on L34, suggesting a higher tolerance to the high dose capsaicin of L34. Although the influence of diets on fecal microbiome patterns [22] and antibiotic resistance of probiotic bacteria [93] are well-known, the data on probiotic resistance against some specific diets is still very less.

To further test an impact of capsaicin tolerance of *L rhamnosus*, probiotics (L34 or LGG) or placebo was administered in healthy volunteers who had spicy foods at least a meal per day. Among these volunteers, the abundance of fecal *Lactobacillus* in placebo group was very less and the probiotics administration at least 7 days was necessary to sustain the fecal abundance which rapidly decreased within 3 days after stop the probiotics. The alterations of fecal abundance of L34 and LGG after administration were not different, despite the higher tolerance against capsaicin of L34 *in vitro*. Perhaps, the dose of capsaicin from the regular chili-containing Thai foods was not high enough to show the difference in abundance of L34 and LGG in feces. More studies of the influence of diets on probiotics are interesting.

## Conclusion

Thai chili extracts and high dose capsaicin induced gut barrier defect through enterocyte cytotoxicity and bactericidal activity-induced gut dysbiosis which were attenuated by *L*. *rhamnosus* probiotics. Although *L*. *rhamnosus* L34 (the Thai-derived probiotics) was more tolerance against 2 mM capsaicin than *L*. *rhamnosus* GG (Caucasian-derive probiotics), the *Lactobacillus* fecal abundance in the healthy Thai volunteers with chili ingestion of both strains of probiotics bacteria was non-different. Further tests on the volunteers with heavy chili ingestion are interesting.

## Supporting information

S1 Fig The non-metric multidimensional scaling (NMDS) based on Thetayc dissimilarity plot of bacterial communities indicates the relational patterns among groups (A) and the bacterial abundance in feces of *Streptococci* and *Butyricicocci* are demonstrated (B-C).
(TIF)
