# Supplementary figures and images for: Lactobacillus rhamnosus attenuates Thai chili extracts induced gut inflammation and dysbiosis despite capsaicin bactericidal effect against the probiotics, a possible toxicity of high dose capsaicin
Source: PLoS One. 2021 Dec 23;16(12):e0261189. doi: 10.1371/journal.pone.0261189 (PMC8699716; doi:10.1371/journal.pone.0261189)

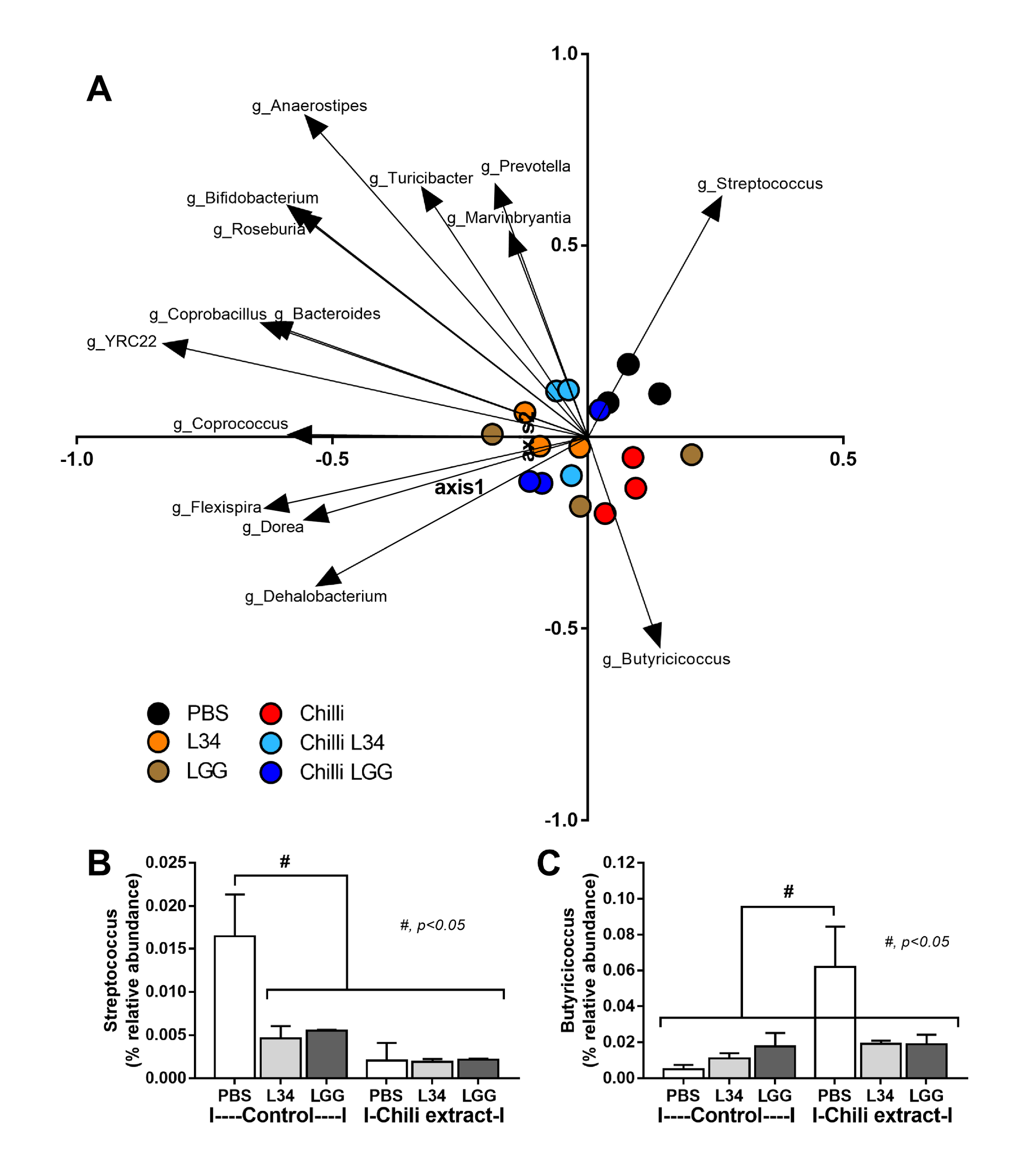

Supplement: S1 Fig — The non-metric multidimensional scaling (NMDS) based on Thetayc dissimilarity plot of bacterial communities indicates the relational patterns among groups (A) and the bacterial abundance in feces of Streptococci and Butyricicocci are demonstrated (B-C). (TIF) [file pone.0261189.s001.tif]
